# Using a Clinical Data Warehouse to Calculate and Present Key Metrics for the Radiology Department: Implementation and Performance Evaluation

**DOI:** 10.2196/41808

**Published:** 2023-05-22

**Authors:** Leon Liman, Bernd May, Georg Fette, Jonathan Krebs, Frank Puppe

**Affiliations:** 1 Chair of Computer Science VI Würzburg University Würzburg Germany; 2 Management und Beratung in der Medizin (MBM) Medical-Unternehmensberatung GmbH Mainz Germany; 3 Service Centre Medical Informatics University Hospital of Würzburg Würzburg Germany

**Keywords:** data warehouse, electronic health records, radiology, statistics and numerical data, hospital data, eHealth, medical records

## Abstract

**Background:**

Due to the importance of radiologic examinations, such as X-rays or computed tomography scans, for many clinical diagnoses, the optimal use of the radiology department is 1 of the primary goals of many hospitals.

**Objective:**

This study aims to calculate the key metrics of this use by creating a radiology data warehouse solution, where data from radiology information systems (RISs) can be imported and then queried using a query language as well as a graphical user interface (GUI).

**Methods:**

Using a simple configuration file, the developed system allowed for the processing of radiology data exported from any kind of RIS into a Microsoft Excel, comma-separated value (CSV), or JavaScript Object Notation (JSON) file. These data were then imported into a clinical data warehouse. Additional values based on the radiology data were calculated during this import process by implementing 1 of several provided interfaces. Afterward, the query language and GUI of the data warehouse were used to configure and calculate reports on these data. For the most common types of requested reports, a web interface was created to view their numbers as graphics.

**Results:**

The tool was successfully tested with the data of 4 different German hospitals from 2018 to 2021, with a total of 1,436,111 examinations. The user feedback was good, since all their queries could be answered if the available data were sufficient. The initial processing of the radiology data for using them with the clinical data warehouse took (depending on the amount of data provided by each hospital) between 7 minutes and 1 hour 11 minutes. Calculating 3 reports of different complexities on the data of each hospital was possible in 1-3 seconds for reports with up to 200 individual calculations and in up to 1.5 minutes for reports with up to 8200 individual calculations.

**Conclusions:**

A system was developed with the main advantage of being generic concerning the export of different RISs as well as concerning the configuration of queries for various reports. The queries could be configured easily using the GUI of the data warehouse, and their results could be exported into the standard formats Excel and CSV for further processing.

## Introduction

### Background

Examinations performed by the radiology departments of hospitals, such as creating X-ray, computed tomography (CT), magnetic resonance imaging (MRI), or ultrasound images, are fundamental for many kinds of clinical diagnoses. Therefore, optimizing the use of radiology is important for any clinician working with it as well as for any patient being examined there. Such optimization has several advantages for the hospital, such as shorter times patients need to stay there as well as the ability to perform more radiologic examinations. It also has advantages for the patient, such as shorter times to wait for the radiology appointment as well as reduced radiation exposure, if unnecessary repeated examinations of the same body region are avoided.

### Objectives

This optimization requires a good overview of the various key metrics of radiologic services and their changes over time. A systematic approach for computing such metrics is building and using a radiology data warehouse. The main requirements for a radiology data warehouse solution are:

- Generic data import from the underlying radiology information system (RIS), for example, via an intermediate data format
- Tools for enriching the basic data with inferred data via a preprocessing step, which allows for more simple and compact queries on the data
- An expressive query language
- A comfortable graphical user interface (GUI) for the query language, including the ability to specify the resulting reports as tables, graphs, or a standard export format for further processing
- An efficient engine for answering queries and generating reports

These requirements are further explained in the following sections.

### State of the Art

The relevance of calculating the key metrics of radiology data [1], as well as the types of metrics, that are most interesting for radiology exports [2,3] has already been described. In addition, the benefits of presenting such metrics in an easily understandable dashboard [4,5] have been explained. Although such solutions have been implemented for many different uses cases [6-9], all of them use a fixed interface to 1 or multiple specific hospital information systems and provide the user with only a fixed selection of predefined calculations. In other systems, the primary goal is to show data from individual patients [10-12], which only allows for a limited amount of filtering and no user-defined queries on the data. Other approaches use a data warehouse [13,14] to unite data from several (still fixed) hospital information systems into a unified representation and therefore allow for various user-defined queries to be executed but are missing a GUI for users to specify their queries and instead have their users either use Microsoft Excel or Structured Query Language (SQL; ISO/IEC Joint Technical Committee 1/Subcommittee 32/Working Group 3) for report creation. For importing data into a clinical data warehouse, more generic solutions exist [15,16] but without an option to calculate additional features during the import. This could make certain types of reports difficult or, depending on the query language of the clinical data warehouse, even impossible to create. These solutions are further discussed in comparison to the developed solution in the final section.

For hospitals whose data have been used during the development of this system, the state of the art for calculating key metrics of their radiology data was to do so manually in Excel. Although this allows for many different reports to be created, it has several drawbacks, which are also discussed in the final section. An intermediate result of this work has already been described [17]. This is described in more detail, together with the improvements in the final result, in the following sections.

### Requirements

#### Generic Data Import Into a Data Warehouse

A radiology data warehouse primarily needs data of the examinations (type of modality, date and time of the request, execution, and documentation of each examination), relevant basic and radiologic patient data, the medical question for the examination as well as the radiologic diagnosis, and information about the radiologic equipment used. Since hospitals use many kinds of RIS, the use of an intermediate data format facilitates the data import and makes it independent of the internal data structure of the RIS. In this project, an Excel (or comma-separated value [CSV]) table was used as an intermediate format, in which the RIS data could be exported and from which it could then be imported into the data warehouse. If a hospital could only export its RIS data into JavaScript Object Notation (JSON) format (a proprietary one or a standard one such as Health Level Seven [HL7] Fast Healthcare Interoperability Resources [FHIR]), the relevant information from this format could also be converted into a table (using an Excel or a CSV file) that uses the structure described in the next section. All the hospitals whose data were used during the development of this system were only able to provide Excel exports of their RIS data.

#### Semantic Preprocessing of the Basic Data

To make queries on the radiology data easier, preprocessing of basic data is useful. Therefore, additional values were inferred from the basic data during the import into the data warehouse. Two types of preprocessing were necessary for this project: The first type was calculations performed by combining information from the basic data. Examples of such precomputed values are the difference between the time when an examination was requested and the time when it was performed as well as the time when the radiologic images were interpreted. This is usually not available in the RIS directly but can be easily computed from the individual time stamps. The second type was standardizations of the basic data. For example, the medical question for the examination could be either available as unstructured text using different wordings or as a hospital-specific code, which must be associated with a readable, standardized description, for example, by using a regular expression during the import. As new kinds of queries are requested, additional data may be required. Because of this, another requirement is the ability to perform an incremental update of the data warehouse with just the new data instead of deleting and reimporting everything that has already been loaded into it.

#### Types of Queries and Query Language

The developed system should be able to support a wide range of different calculations. The calculations requested by the hospitals with whose data the system has been used so far could be separated into 5 different categories, which are described here:

- Patients, appointments, and examinations per modality: The most common metric was the number of patients, radiology appointments, and examinations in the radiology department for each modality. Additionally, these numbers were separated between inpatients and outpatients, the department of the hospital requesting an examination, the region of the body that was examined, or the shift during the day in which the examination took place. All these numbers were used to provide a general overview of the use of the radiology department.
- Use of radiologic devices: A radiology department usually has many different devices for different modalities as well as often multiple devices for a single modality. To better distribute examinations and clinicians on these devices, their use is 1 of the requested calculations. The metrics for this use included the number of examinations and patients per device. Furthermore, the time for each examination as well as the vacancy between examinations were evaluated.
- Length of a patient’s stay in the hospital: Depending on the disease, different lengths of stay in the hospital are necessary. To evaluate whether patients were staying longer in the hospital than expected, which results in a reduced capacity for new patients, the actual stay times were compared with the ones suggested by clinical guidelines.
- Waiting times: Short waiting times are in the interest of both the patient and the clinician requesting a radiologic examination. Therefore, for each modality, the time between the request of an examination, the actual appointment in the radiology department, and the availability of the clinical findings after the examination were calculated and compared.
- Multiple examinations for the same question: To find the answer to a specific medical question, in many cases, 1 kind of radiologic examination works best. If such radiologic examination is performed directly by an experienced radiologist (who also verifies whether the requested examination makes sense for the medical question), the chances are high that only 1 examination is necessary to answer the medical question. However, if for 1 medical question, multiple examinations with the same or with different modalities are performed, the patient has increased radiation exposure and fewer radiology appointments are available for other patients. To measure this, first, all the different sequences of modalities for different kinds of medical questions were calculated. Afterward, the number of patients with such sequences were counted and compared. In addition, the total time for which a patient with such repeated examinations stays in the hospital was evaluated.

The query language used by the developed system must be able to support these kinds of queries as well as additional ones requested by the hospitals. This is also important for evaluating possible ways in which any of these metrics can be improved. For example, unnecessary multiple examinations can perhaps be explained by too few available devices for the modality recommended for a question or by missing staff to operate a device. To verify whether the measures taken are successful, the query language must also be able to analyze the change in the metrics over time.

A common set of queries for data saved in the same way furthermore allows for an easy comparison of the calculated number between different hospitals. In addition, as none of the mentioned categories depends on a specific hospital, all these calculations can be performed for any hospital (even in different countries) if it is able to provide the necessary data from its RIS.

#### A Comfortable GUI for the Query Language and the Result Specification

Although the query language should be usable in textual form, a GUI is also required to create queries in a graphical way and automatically create the corresponding textual queries. As with the query language, the GUI should also allow for the layout of the requested report to be specified. The results of queries should be shown to the user as a table or as a graph. Furthermore, the results should be exportable into the standard formats Excel and CSV so that they can be further processed.

Furthermore, the GUI should make the system (with a limited amount of training) usable by the clinicians themselves and therefore should not require any knowledge of computer science.

#### Efficiency Requirements

Importing data into the data warehouse as well as creating reports using the query language on these data both should happen in a reasonable amount of time. For the initial import, the tool should not need longer than a few hours, and for querying the data, most of the queries should return their results in about 1 second, while more complex queries should not run for more than a few minutes. These requirements are necessary so that a user can quickly start to use the system and, while using it, easily try different variations of a query without a long waiting time for each result.

## Methods

### Ethical Considerations

In this paper only retrospective, pseudonymized patient data for patients with age groups below and above 18 years with a few attributes only about their radiologic examinations were used (dates, modality, device, localization, radiologic query, boolean values for insurance [statutory or private], boolean values for the type of stay in the hospital [inpatient or outpatient]). De-pseudonymization of the data was not possible for the authors of the study. Therefore, no ethics approval was necessary.

### Concept

#### Processing Radiology Data for Importing It Into a Data Warehouse

The data from the RIS of the hospitals were provided to the tool as an Excel, CSV, or JSON file, in which each row represents a single examination. Each column in this file is 1 attribute, and the names of these attributes are written in the first row of the file.

To map these columns to attributes in the structure of the data warehouse, a configuration file (using Excel or CSV as well) was used. This file contained 1 row for each attribute and specified the name, identifier, and data type to use (eg, numbers or texts) when importing them together with the concrete values into the data warehouse. The columns containing the required metadata (eg, identifiers) must be specified in this configuration file as well.

As mentioned in the previous section, some values for the requested reports must be calculated based on the exported RIS data. To do this, several options were offered. Additional columns were added to the RIS export performing the calculations. These were then imported into the data warehouse like any other column in the RIS data by specifying them in the configuration file. The configuration file also provided an option to replace textual values with other values, which could, for example, be used to replace an abbreviation in the RIS export with a longer form. For more advanced calculations, several programming interfaces were offered and could be implemented for any value requiring such a calculation.

All the values from the RIS, together with the calculated values, were then saved to the data warehouse, and an index was created on them for increased query performance.

#### Creating Reports

As soon as all the needed values were saved in the data warehouse, queries on these data were run to calculate and create the requested reports. For this purpose, a query language was used to define the structure of the report. This was done by first specifying attributes to be queried as well as constraints on the values of these attributes. In the next step, these attributes were combined with the logical operators “and”, “or,” and “not.” These single attributes or groups of attributes were then used to specify the rows and columns of the requested report. For every combination of attributes in each row and each column, a query was created, resulting in the cells of the report. If additional constraints on all cells were required, other attributes were used to specify filters. Finally, the query language specified what type of count (examinations, appointments, or patients) should be returned. All this was either specified in textual form or graphically using the GUI of the data warehouse. To create a report, the query for each cell was run on the data and, depending on the configuration, the number of examinations, appointments, or patients was returned.

By using a query language like this, it is easily possible to run modifications of a query, which is further simplified by the ability of the data warehouse to save a query and load it again later.

After a query was configured and executed, the interface of the data warehouse showed the results as a table and provided the option to export this table into the standard formats Excel and CSV. The results of some predefined queries were also shown as graphics.

### Implementation

#### The Clinical Data Warehouse PaDaWaN

PaDaWaN (short for Patient Data Warehouse Navigator) [18] was used as the clinical data warehouse. Its core is a database containing all the used medical information and a separate index to increase the speed of queries on the data. To specify the queries, PaDaWaN uses its own query language as well as its own web interface. Furthermore, it provides the ability to export and save query results. All these parts are described in more detail in the following sections.

##### Database Structure

**Figure 1:**
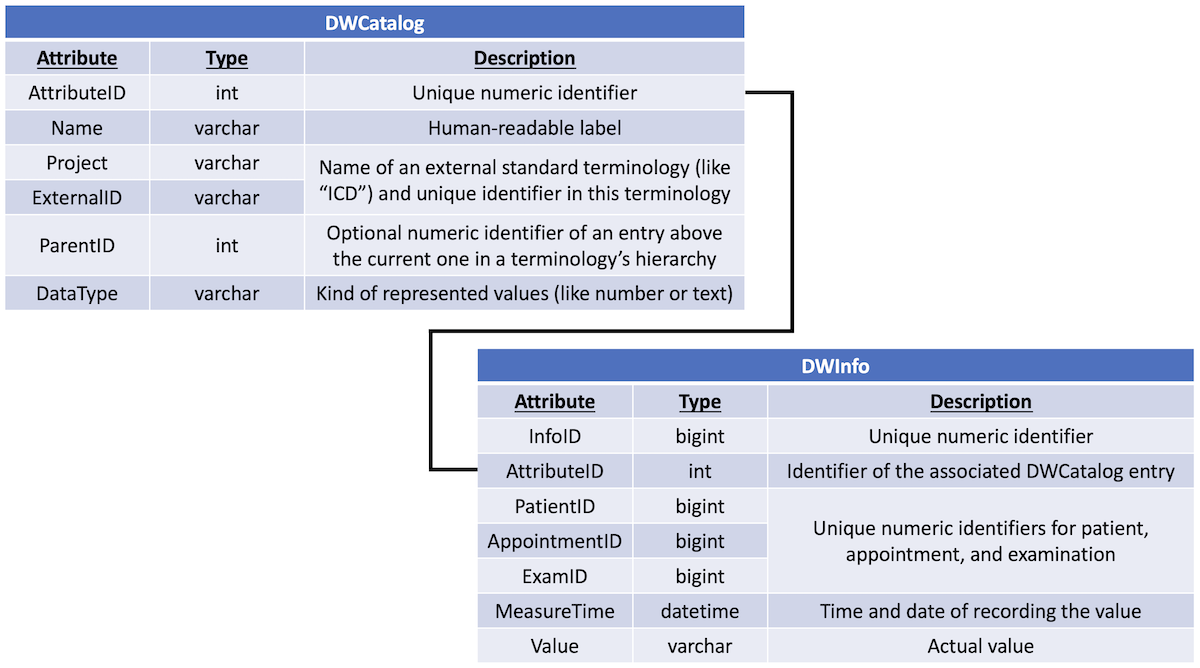
Structure of the 2 main tables in PaDaWaN’s database containing all the possible types of information (DWCatalog) and the information itself (DWInfo). ICD: International Classification of Diseases; PaDaWaN: Patient Data Warehouse Navigator.

PaDaWaN stores its data in a database, which could be either a Microsoft SQL [19] or a MySQL [20] database. The table structure is based on the entity attribute value model [21]. It consists of 2 main tables, as shown in Figure 1.

The first table (DWCatalog) contains a catalog of all the possible types of information in PaDaWaN. This could represent, for example, different types of diagnoses or laboratory measurements. Each entry in this table is uniquely identified by a numeric AttributeID as well as by the combination of ExternalID (the ID in the terminology defining the entry, such as, the International Classification of Diseases [ICD] code [22] I50) and Project (the name of the whole terminology, such as “ICD”). AttributeID is automatically generated by the database and is only valid for a single installation of the system. As further explained later, only the combination of ExternalID and Project is used to identify entries from this table in a query, and therefore, only this combination must be unique among different systems if the same query should be used for all of them. For easier usage in the PaDaWaN interface, every entry has a readable name (eg, “heart failure”). The ICD terminology, for example, uses a hierarchal structure. To save this or any other hierarchy among the attributes, the ParentID field is used and contains the AttributeID of the entry, that is, the parent of the current entry. The kind of data (eg, Boolean, number, or text) that could be saved for an entry is specified with the DataType field.

In the DWInfo table, all the concrete patient data are saved. Each entry in this table is uniquely identified with an automatically generated numeric InfoID and is associated with a type of information from the DWCatalog table by its AttributeID. With the other ID fields, each entry in this table is also associated with a patient, appointment, and examination. The time and date on which a value has been recorded is saved in the MeasureTime field. The actual value (eg, the content of a patient’s discharge letter) is stored as text in the Value field.

##### Index Structure

**Figure 2:**
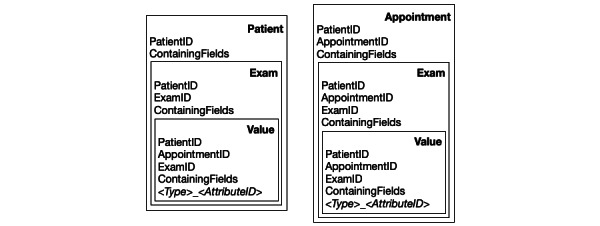
Document structure of PaDaWaN’s Solr index using nested documents for examinations and values in separate parent documents for patients and appointments. In addition to the numeric identifiers for patients, appointments, and examinations under ContainingFields, all AttributeIDs of all values inside a document and its children are saved. The values themselves are stored in dynamic fields named with a combination of their type and AttributeID. PaDaWaN: Patient Data Warehouse Navigator.

To increase the speed of queries on the data in PaDaWaN’s database, it was indexed with Apache Solr [23]. Solr saves data in documents, and the schema used for PaDaWaN is shown in Figure 2.

PaDaWaN offers the ability to search for data on 3 different levels: patients, appointments, and examinations. If a search is conducted on any of these levels, all patients/​appointments/examinations should be found, containing all the requested combinations of attributes and values. To accomplish this in Solr, PaDaWaN uses Solr’s feature to store documents nested in other documents. As shown in Figure 2, a document is created for each patient and each appointment. Another document is created for each examination and is stored inside the patient and appointment documents. Finally, for every value, another document is created and stored inside both examination documents. Although this approach requires more disk space as each value is saved twice, this greatly increases the speed of queries being run on the patient level compared to a document structure, where the appointments are nested inside the patient documents. Each document contains all the available IDs, as described in the previous section. Additionally, all of them contain a field named ContainingFields, which stores the AttributeIDs of all values contained in the current document. This allows a query to restrict the number of top-level documents it must search on for concrete values of the attributes. These values are stored in a field in the value document, whose name is generated by combining the attribute’s type with its ID.

##### Query Language

**Figure 3:**
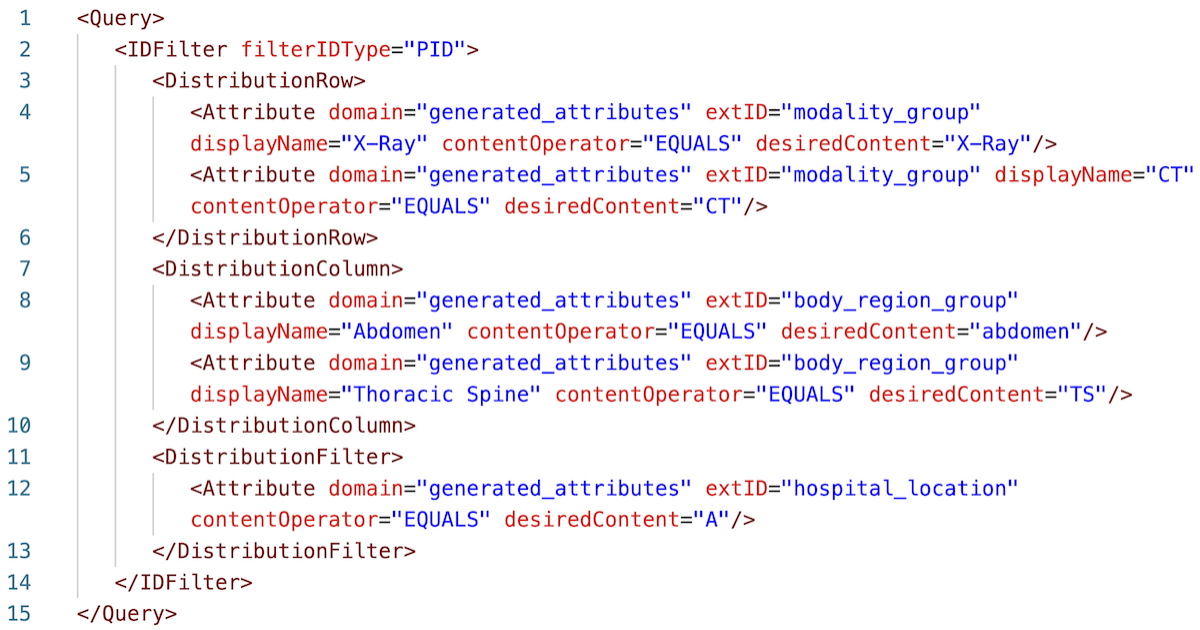
Sample query in PaDaWaN’s query language MXQL. This query returns counts of patients (specified with the filterIDType “PID”) for each combination of attributes specified as DistributionRows and DistributionColumns. In this example, the first combination would be all X-ray examinations of the abdomen. DistributionFilter restricts all the combinations to patients from hospital A. MXQL: Medical XML Query Language; PaDaWaN: Patient Data Warehouse Navigator.

**Figure 4:**
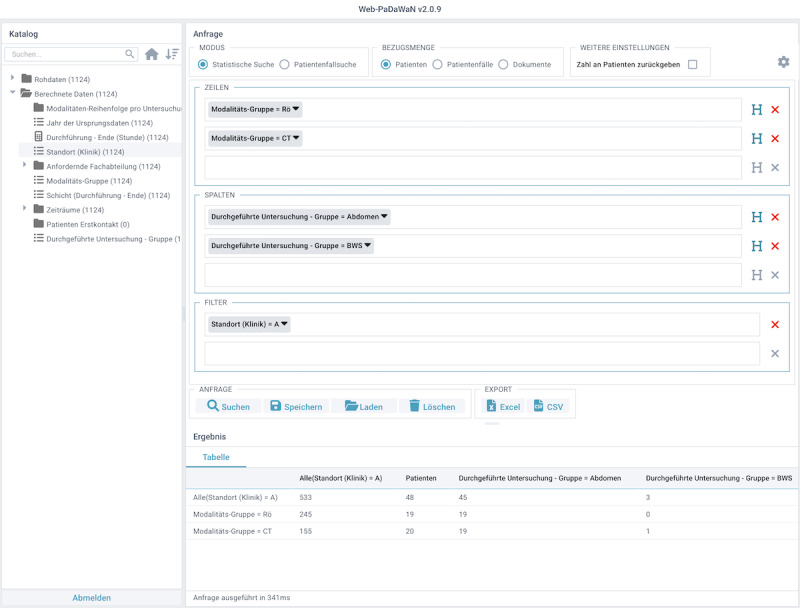
Web interface of PaDaWaN with the query from Figure 3. On the left side, the catalog of available attributes is shown and can be hierarchically expanded as well as searched. On the top of the right side, the query itself can be configured by dragging items from the catalog to create rows (Zeilen in German), columns (Spalten in German), and filters. With the 3 radio buttons in the top middle, the kind of IDs to be counted can be specified. Although meaning something else in German in this project, the buttons from left to right are used for patients, appointments, and examinations. The row of buttons in the middle are used to execute a query (Suchen in German) as well as to save and load queries (Speichern and Laden in German, respectively) and to export their results. After executing a query, the bottom right of the GUI shows its result (Ergebnis in German). The remaining buttons were not used for this project. The query shown here creates rows for X-ray (Rö: short form in German) and CT scan (CT: short form in German) examinations and columns for examinations of the abdomen and the thoracic spine (BWS: short form in German). The filter then restricts everything to just examinations from the hospital (Klinik in German) A. The remaining elements of the GUI were not used for this project. CT: computed tomography; GUI: graphical user interface; PaDaWaN: Patient Data Warehouse Navigator.

**Figure 5:**
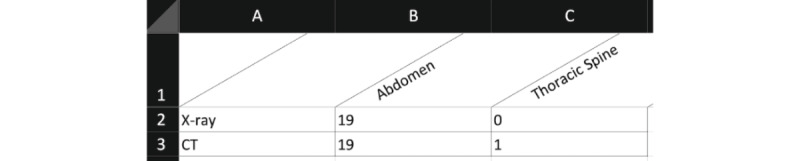
PaDaWaN Excel export for the query from Figures 3 and 4, where the number of patients with different kinds of radiology examinations (as rows) is counted for multiple regions of the human body (as columns). CT: computed tomography; PaDaWaN: Patient Data Warehouse Navigator.

To specify the structure of the requested tabular result, PaDaWaN uses its own query language called Medical XML Query Language (MXQL). In the following example, the result table contains 2 rows and 2 columns. The rows contain 2 different types of modalities (X-ray and CT scan), and the columns contain 2 regions of the human body (abdomen and thoracic spine). For each combination of a row and a column, the number of matching patients from hospital A is returned. In the first cell, for example, the number of patients who undergo an X-ray examination of the abdomen is counted. This query is shown in MXQL in Figure 3 and in PaDaWaN’s GUI in Figure 4. The result in Excel can be seen in Figure 5. This is a simple example used to explain the query language, PaDaWaN’s GUI, and its export capabilities, and the results shown in Figure 5 may also be retrieved directly from an RIS (depending on its capabilities).

Each query in MXQL must contain at least the following 2 elements: Query and Attribute. Query is the root XML element and contains the whole rest of the query. Attribute contains information about the catalog entry whose values should be queried. To identify this catalog entry, Attribute uses the “domain” and “extID” properties, which map to the Project and ExternalID columns of the DWCatalog database table described before. The remaining elements of the query are optional and used for more complex queries. In the example query shown in Figure 3, the Attribute elements are further constrained to only match specific values for the catalog entries. This is done with the contentOperator and desiredContent properties, where desiredContent contains a value to be matched and contentOperator the way it should be matched. The IDFilter element is used to specify on which level all the attributes in the query should be combined. In the example in Figure 3, this is set to “PID,” which means that all the attributes must have the same PatientID and that the number of matching patients should be returned by such a query. The last remaining elements of the query are DistributionRow, DistributionColumn, and DistributionFilter. They are used to return counts of multiple combinations of Attributes in a single query. Each DistributionRow becomes a row in the created result, and similarly, each DistributionColumn becomes a column. The DistributionFilter can be used to apply further constraints on all the combinations of rows and columns. Finally, the displayName property of the Attribute element can be used to provide a name for the created rows and columns. Not shown in the example is the ability of MXQL to combine multiple attributes with the logical combinations “and” and “or,” which could even be nested inside another combination. MXQL also allows for the logical operator “not” to be added to any attribute.

Here, only the MXQL features used for this project are described. A complete documentation of this query language (in German) can be downloaded from PaDaWaN’s website [24].

##### Web Interface

PaDaWaN has its own graphical web interface allowing a user to search the available attributes, graphically configure a MXQL query, and preview the result table. The interface with the MXQL query from Figure 3 looks like Figure 4.

On the left side of the interface, the content of PaDaWaN’s DWCatalog table (explained before) is shown and can be hierarchically expanded and searched. With the 3 radio buttons in the top middle of the GUI, the level on which all the attributes in the query should be found (for this project, patients, appointments, or examinations) can be configured. Via drag and drop, any attribute from the catalog can be placed in any section of the query to configure either rows (*Zeilen* in German), columns (*Spalten* in German), or filters (like that explained in the previous section). Finally, a configured query can be run by pressing the Search button (*Suchen* in German). With the Save and Load buttons (*Speichern* and *Laden* in German, respectively), a configured query can be saved and any saved query can be loaded. The next 2 buttons provide the option to export a queried result in either Excel or CSV format. The bottom of the right half of the GUI shows the tabular result (*Ergebnis* in German) created after running a query. By clicking any of the attributes in the query on the right side of the GUI, a dialog box appears, where, for example, the value of an attribute that should be counted can be configured. In this example these values are *Rö* (short in German for X-ray), *CT*, *Abdomen*, *BWS* (short in German for thoracic spine), and *A* (for the name of the hospital; *Klinik* in German). All the remaining buttons were not used for the queries in this project.

##### Export of Query Results

PaDaWaN also offers an option to export query results in either Excel or CSV format using the Excel or CSV button, respectively, in Figure 4. When the query has finished, an Excel or a CSV file is created and offered as a download. When running the query shown in MXQL in Figure 3 and in the GUI in Figure 4, the Excel export looks like that in Figure 5.

As configured in the query, each row is a different kind of radiologic examination, and each column contains a different region of the human body. As the query was configured to return the number of matching patients, the first number means that in this (small and artificially generated) data set, 19 patients underwent an X-ray examination of the abdomen.

#### Export of the Radiology Data

**Figure 6:**
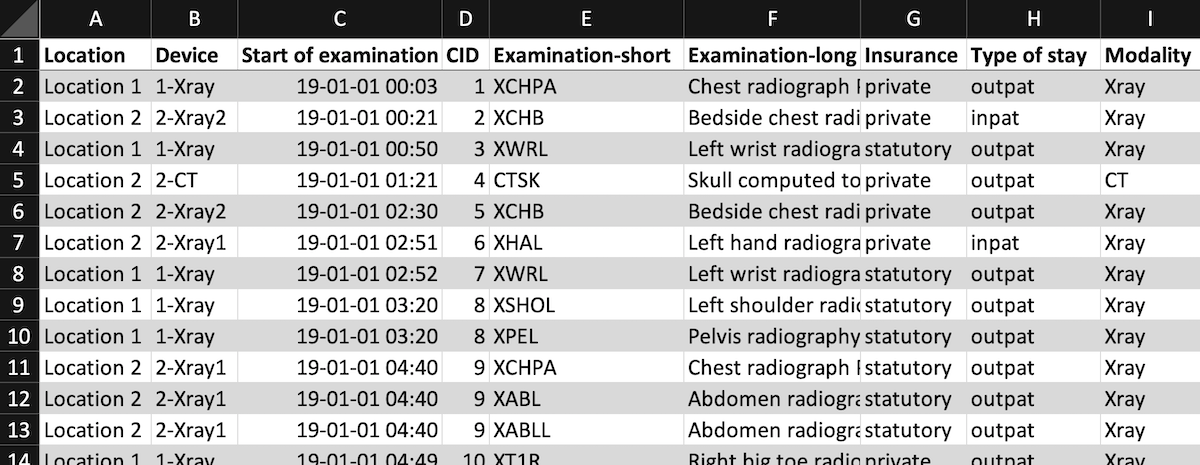
Sample of an RIS Excel export containing information about 1 examination in the radiology department per row and 1 attribute per column. CID: Case identifier; CT: computed tomography; RIS: radiology information system.

For the developed system, data from a hospital’s radiology department are needed. The system should be usable by many different hospitals with many kinds of RISs. Therefore, Excel, CSV, and JSON are used as the formats in which the RIS data can be exported and then imported from this file into PaDaWaN. As mentioned in the Introduction section, if a hospital is only able to provide its RIS data as a JSON file, this can also be transformed into a table and then saved as either an Excel or a CSV file. A part of an RIS Excel export is shown in Figure 6.

Exporting uses a simple structure, where each row represents a single examination of a patient and each column contains 1 attribute with information about the examination. The title of the attribute must be given in the first row. The only required pieces of information are the ID of the patient’s stay in the hospital, the start date and time of the examination, and the modality performed. All the remaining attributes could be different for each hospital, and the way they are imported into PaDaWaN is explained in the next section.

All the IDs that were used for this project had already been pseudonymized during the RIS export.

#### Import of the Radiology Data Into the Data Warehouse

The RIS export, as described in the previous section, was imported into PaDaWaN using the following steps:

- Step 1: A configuration file is created to specify the mapping of the RIS export columns to PaDaWaN catalog entries.
- Step 2: Using this configuration file, the data in the RIS export are converted to PaDaWaN database entries.
- Step 3: Additional precalculations are performed on the RIS data using an interface provided, and the results are saved in PaDaWaN’s database as well.
- Step 4: A Solr index is created on these data.

**Figure 7:**
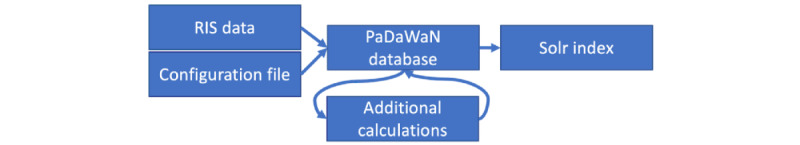
Overview of the process for importing radiology data into the data warehouse. First, a configuration file is created and used to import the exported RIS data into PaDaWaN’s database. On these data, additional precalculations are then performed. Finally, a Solr index is created for all the data in PaDaWaN’s database. PaDaWaN: Patient Data Warehouse Navigator; RIS: radiology information system.

An overview of this process is shown in Figure 7. Each step is described in more detail later.

##### Configuration File to Map the Radiology Data to the Data Warehouse’s Structure

**Figure 8:**
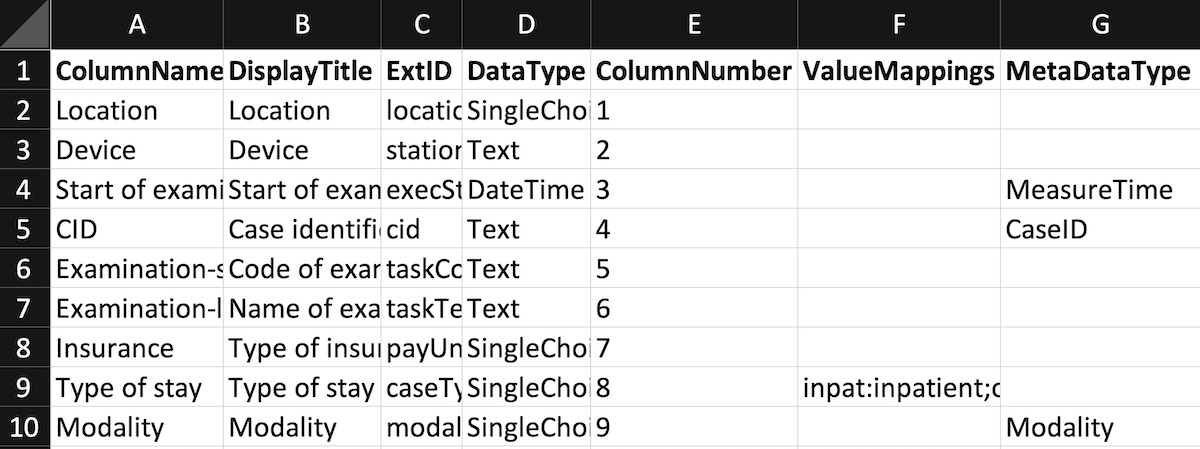
Sample of an Excel configuration file to specify the mapping between an RIS export and PaDaWaN’s data structure. The ColumnName and ColumnNumber columns must match a column in the RIS export. The DisplayTitle, ExtID, and DataType columns are mapped to the corresponding columns in PaDaWaN’s DWCatalog table. With ValueMappings, column abbreviations in the RIS export can be mapped to their longer form. The final column is used to specify which RIS column contains which type of metadata. CID: Case identifier; PaDaWaN: Patient Data Warehouse Navigator; RIS: radiology information system.

An Excel (or CSV) configuration file was used to specify the mapping of the columns in the RIS export to the data structure of PaDaWaN. A configuration file for the data in Figure 6 would look like that in Figure 8.

The first row of this file must always look like that shown in Figure 8. Each of the following rows represents 1 column from the RIS export. If any of these columns should be ignored, they can be left out. The columns of the configuration file are used for specifying the PaDaWaN catalog entry that should be created (with the name from the DisplayTitle column and ExtID and DataType being directly used for database columns with the same names). The DataType “SingleChoice” is used for textual values with only a limited number of possible options (eg, modality). The ColumnName and ColumnNumber columns are used to identify a column in the RIS export. The ValueMappings column can be used to map abbreviations or codes in the RIS data to more readable names. Finally, the MetaDataType column is used to specify which columns contain which type of identifiers, the time an examination was performed, and the modality.

##### Import Process of the Radiology Data Using the Configuration File

When starting the import of the RIS export, first, the configuration file, as explained before, is read and then all the columns specified in the rows of the configuration file are imported into PaDaWaN.

For this, first, an entry in PaDaWaN’s DWCatalog table is created with the values from the configuration file. To import concrete values from any column in the RIS export into PaDaWaN’s DWInfo table, some metadata are required: PatientID, AppointmentID, and ExamID, as well as MeasureTime. These are specified with the MetaDataType column in the configuration file.

With the catalog entry and the metadata, each value in each column of the RIS export was saved into PaDaWaN’s database.

##### Calculating and Importing Additional Values Based on the Radiology Data

As some calculations are not possible with PaDaWaN’s query capabilities or would require complex queries, several interfaces (written in Kotlin [25]) are provided to specify additional calculations that should be executed during the RIS data import. Initially, for all these interfaces, the properties of the PaDaWaN catalog entry that should be created must be provided. Additionally, the RIS column names required for the calculation must be specified. The provided interfaces can then be used to either specify calculations that should occur for each examination (eg, calculating the shift during a day in which an examination was performed) or once for all examinations (eg, to calculate sequences of examinations that have been performed for a single patient and for the same medical question with 1 or multiple modalities).

During the execution of all the implementations, the catalog entry specified by each implementation is created and the implemented methods to calculate the values and save them to the database are called.

##### Creating an Index on All Imported and Calculated Values

The last step during the import process of the RIS data is the creation of a Solr index on the data from PaDaWaN’s database. For this purpose, all the values are fetched from the database and documents in the structure described before are created. These documents are then sent to Solr, which creates its index on them.

#### Incremental Updates of the Data Warehouse for New or Updated Radiology Data

The process of importing all the RIS data into PaDaWaN as well as creating a Solr index on it takes some time (shown in the next section). During the work on this project, additional calculations on the RIS data, updates on existing calculations, and additional information from the RIS were needed in many cases. The whole process described in the previous section could be run again, which resulted in most values being imported or calculated again, although they did not change.

Therefore, a separate configuration file could be given to the importer, containing just the names of the attributes from the RIS export or from the implemented interfaces, that had to be processed. When using this option, just the columns and calculations of these attributes are processed and saved to the database. Afterward, Solr’s ability to perform atomic updates [26] is used. In this way, the whole documents do not have to be created and indexed again, but instead, only small parts for the updated or added attributes are deleted and then added with the new values to the existing documents.

Another possibility for new radiology data would be data from new patients. In this case, the additional data can be exported from the RIS into a separate file and then the whole import process described before can be run for just this file so that only the new data are added to the database and the index and no processing of the existing data must be done again. If a near-real-time evaluation of the data is requested by a hospital, this process can also be run immediately any time new data are added to the RIS.

#### Performing Calculations on the Data Using the Data Warehouse and Exporting Their Results

After the RIS data and any additional calculations on them are saved to and indexed by PaDaWaN, PaDaWaN’s web interface is used to create and run queries on these data. The usage of the interface as well as the query capabilities have already been described in the section on PaDaWaN before.

The process of creating a new query involves first specifying all the attributes for rows and columns whose combinations should be counted in the result. Optionally, additional filters can be configured for all these combinations. Next, the user chooses whether the number of matching patients, appointments, or examinations should be returned. Finally, the query is run (the matching MXQL query is automatically created by the web interface), and its result is either shown directly in the GUI or is exported to an Excel or a CSV file.

For reusing queries, PaDaWaN also provides an option to save and load queries.

PaDaWaN’s web interface uses a REST-based interface, which can also be used directly without the GUI. To do so, the query must be created as an MXQL string and can then be sent to the interface. When PaDaWaN has finished the execution of the query, the result can be received in JSON format or as an Excel or a CSV file.

#### Presentation of Results Calculated by the Data Warehouse

**Figure 9:**
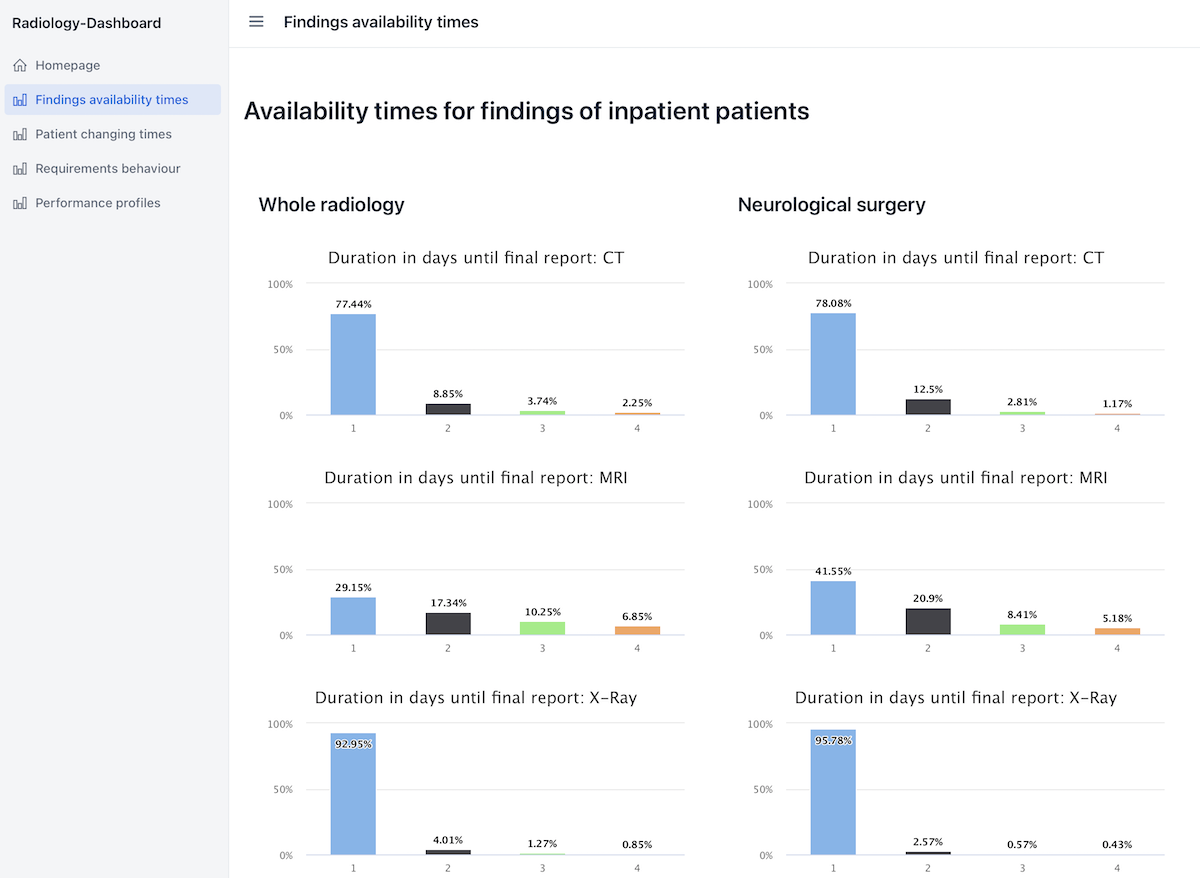
Simple web dashboard with graphics for the 4 most common types of calculations on the radiology data of the hospitals involved in this project. The graphics in this figure show what percentage of findings for a radiological examination is available in up to 1, 2, 3, or 4 days. This is given for the 3 most common modalities and is compared between examinations in the whole radiology department and only the ones requested by neurological surgery. CT: computed tomography; MRI: magnetic resonance imaging.

As PaDaWaN allows for exporting of results into the standard formats Excel and CSV, these results can be easily imported by many different tools to perform further calculations or to create graphics. To present the 4 most common types of calculations for the hospitals involved in this project as graphics, a simple web dashboard was created and is shown in Figure 9.

The example calculates for all inpatients the percentage of findings for 3 modalities that has been available for 1, 2, 3, or 4 days after the examination in the radiology department. These numbers are further compared between the whole radiology department and just examinations that have been requested by the neurological surgery department.

To calculate these numbers, matching PaDaWaN queries were created and saved. When opening this dashboard, the queries were loaded and executed, and the numbers were extracted from PaDaWaN’s result table.

## Results

### Technical Evaluation

In the following sections, details about the used data themselves as well as about the import and report creation process are presented.

#### Used Data

The developed system was tested with RIS exports from 4 different hospitals from different regions of Germany. Some of these data are shown in Table 1.

**Table 1:** Information about the used radiology exports of 4 different hospitals from Germany.

Details	Hospital A	Hospital B	Hospital C	Hospital D
Hospital sites, n	1	2	2	3
Time of data	2018	2019 to September 2020	2018 to June 2021	2019 to 2021
Patients, n	13,603	125,732	N/A^a^	N/A^a^
Appointments, n	28,886	384,186	307,174	241,148
Examinations, n	52,542	487,474	599,481	296,614
Values imported, n	2,50,001	11,650,688	15,014,221	5,339,024
Values generated, n	555,859	18,848,459	8,151,85	2,974,740
RIS^b^ export size (MB)	14.9	75.7	56.1	21.7

^a^N/A: not applicable. The data from hospitals C and D contained no patient identifier, so the number of patients could not be specified.

^b^RIS: radiology information system.

The data were provided as an Excel export from the RISs of the hospitals. The last 3 hospitals had multiple radiologic sites in different cities. In addition, the time for which the data were exported was different, ranging from 1 year for the first hospital to 3.5 years for the third one. Only in the exports of the first 2 hospitals was a (pseudonymized) patient identifier included, so the number of patients could only be calculated for these 2 hospitals. Each value in a single cell of the RIS exports was imported into the data warehouse, and their number is specified in Table 1. For comparison, the number of values that were generated during the import is also specified. Numbers related to the import process are presented in the next section. Finally, in the last row, the size of the Excel files exported from the RIS is shown.

#### Process of Importing the Radiology Data Into the Data Warehouse

For the data of all 4 hospitals, a separate virtual machine (running in the internal network of the university of Würzburg) was created, and each of them was configured with 4 CPUs and 32 GB of RAM and stored on a solid-state drive (SSD). Inside of these machines was installed Ubuntu 20.04.4, together with MySQL 8.0.28, Java 11.0.14, and Solr 8.11.1. PaDaWaN’s web interface was run on an Apache Tomcat [27] 10.0.18 server. On these virtual machines, the RIS exports were imported into PaDaWaN, resulting in the numbers shown in Table 2, which are discussed in the next section.

For 3 of the hospitals (the ones with data from multiple sites and years), the RIS export was provided as several Excel files, which were imported 1 by 1. Their number is specified in the first row of the first section of Table 2. After processing all files, the Solr index creation began.

The next row of Table 2 shows the total time needed for loading the RIS exports and saving their values to PaDaWaN’s database. The number of attributes in the RIS export is specified in the last row of the first section.

**Table 2:** Numbers measured while importing radiology exports into the data warehouse.

Details		Hospital A	Hospital B	Hospital C	Hospital D
**RIS data import**					
	Imported files, n	1	4	8	3
	Import time (hours:minutes:seconds)	0:02:22	0:18:11	0:21:06	0:07:07
	Imported attributes, n	45	26	11	18
**Additional calculations**					
	Calculations, n	10	14	9	8
	Time for each calculated attribute (seconds), mean (SD)	4.7 (2.9)	23.0 (30.2)	9.3 (2.3)	11.7 (8.0)
	Total time (hours:minutes:seconds)	0:00:47	0:21:31	0:11:11	0:04:40
**Index and database**					
	Solr index creation time (hours:minutes:seconds)	0:04:02	0:31:28	0:16:49	0:12:06
	Time of the total process^a^ (hours:minutes:seconds)	0:07:12	1:11:15	0:49:13	0:23:57
	Database size (GB)	1.3	15.8	11.4	3.7
	Solr index size (GB)	1.4	15.1	2.2	3.8
**Incremental updates**					
	Examinations per day, mean (SD)	144 (61.8)	763 (288.5)	469 (200.3)	271 (222.3)
	Time for adding these examinations^b^ (hours:minutes:seconds)	0:00:18	0:01:29	0:01:00	0:00:28

^a^Total time for importing all attributes, calculating additional ones, and creating the Solr index.

^b^Time for incrementally adding just this average number of examinations per day.

In the second section of Table 2, numbers related to the additionally performed calculations are shown. These are the number of calculated attributes, the average time needed to calculate and save them to the database, and the total time for calculating and saving all these values.

The second-to-last section of Table 2 starts with the time needed to create a Solr index of all the imported values as well as the time needed for the whole import process of each hospital. In the last 2 rows, the size of the created database and Solr index is specified.

In the final section of Table 2, additional numbers related to the ability of the developed system to perform incremental updates are shown. Therefore, the average number of examinations per day for each hospital was calculated and then the time was measured to incrementally add just this number of examinations (along with additional calculations on them) to PaDaWaN’s database and index.

#### Creating Reports on the Radiology Data With the Data Warehouse

Many reports were created using the data of all 4 hospitals, depending on the requirements of each hospital. Three reports of different complexities, which were requested by most of the hospitals and were possible with the data provided by all of them, were created to show the time PaDaWaN needed to calculate those reports and export them as an Excel file. For each report, the number of matching examinations was restricted with MXQL to only include the data of 1 year. In all 3 reports, the 4 most common types of modalities for the hospitals (X-ray, CT, MRI, and ultrasound) were used. The following reports were created:

- Report 1: Number of examinations performed for the 4 modalities (as rows of the query) and for the types of stay in the hospital (inpatient or outpatient, as columns of the query)
- Report 2: Number of examinations performed for the 4 modalities (as rows of the query) and for the different hours of the day (from 8:00 a.m. to 7:00 p.m., as columns of the query)
- Report 3: Number of examinations requested by all the different organizational units of each hospital (as rows of the query) for the 4 modalities (as columns of the query)

The numbers related to the creation of these reports are shown in Table 3 and are discussed in the next chapter.

One Solr query was created for each possible row-column combination, which is why the number of executed Solr queries for each report equaled the number of rows multiplied by the number of columns. These numbers were identical for all 4 hospitals in the first 2 reports, as they used the same rows and columns. In the last report, 1 row was created for each organizational unit of the hospital, resulting in different numbers of rows for each hospital.

Table 3 also shows the average time in milliseconds Solr needed for each single query, as well as the total time to run all the Solr queries and export their results as an Excel file.

**Table 3:** Numbers related to the process of creating 3 reports of different complexities with the data warehouse.

Report and details		Hospital A		Hospital B		Hospital C	Hospital D
**Report 1**							
	Rows, n		4		4	4	4
	Columns, n		2		2	2	2
	Solr queries, n		8		8	8	8
	Time^a^ (ms), mean (SD)		79.5 (111.6)		185.3 (264.0)	90.3 (117.3)	95.3 (180.7)
	Total time		645 ms		1 s 489 ms	728 ms	767 ms
**Report 2**							
	Rows, n		4		4	4	4
	Columns, n		12		12	12	12
	Solr queries, n		48		48	48	48
	Time^a^ (ms), mean (SD)		18.3 (7.7)		67.8 (23.3)	22.6 (12.4)	21.8 (13.7)
	Total time		904 ms		3 s 275 ms	1 s 110 ms	1 s 69 ms
**Report 3**							
	Rows, n		48		804	396	2054
	Columns, n		4		4	4	4
	Solr queries, n		192		3216	1584	8216
	Time^a^ (ms), mean (SD)		11.6 (4.2)		25.1 (3.2)	9.1 (3.4)	9.8 (3.3)
	Total time		2 s 315 ms		82 s 233 ms	15 s 201 ms	84 s 758 ms

^a^Average time for the execution of each Solr query.

#### Comparison With the Creation of Reports Directly in Excel

Before using the developed tool, all 4 hospitals created such reports directly in Excel. To evaluate possible improvements compared to the report creation in Excel, this manual process was performed for new reports of different complexities with the largest data set (of hospital B) with the data of 1 year.

When the reports are created directly in Excel, nothing needs to be imported. Nevertheless, to simplify the calculations on the data, all the RIS exports of the considered year were combined into a single Excel file. The calculations otherwise executed during the import process were performed directly in Excel by using Excel formulas in new columns. As all these calculations were executed on each opening of the Excel file, all the RIS data, together with the calculated values, were then copied to another Excel file so that working with the data was faster.

The reports themselves could be created directly in Excel in many ways. If just single numbers are required, Excel’s built-in filter capabilities can be used. To create the reports for this evaluation, Excel formulas were used to define the value of each cell. These formulas were then copied to all the other cells, and their restrictions were adapted according to each row and column of the report that had to be created.

The results of this comparison are discussed in the next section.

### User Feedback

Because the usage of the developed system consisted of various reports requested by the participating hospitals, their feedback was evaluated by describing the requests that could and that could not be created on the data provided by them.

In general, the requests could be divided into those of interest to all hospitals and special requests by an individual hospital. Of general interest was, for example, the number of patients, appointments, and examinations; the use of devices; repeated examinations of the same body region; and the waiting time for an examination. Specialized reports were mainly created for hospital B, which has the largest radiology department among the participating hospitals. The concrete reports that were created for each hospital are listed next.

#### Reports for Hospital A

- Specifically for patients with multiple myeloma or a hepatocellular carcinoma the number of patients for each modality and quarter of the year as well as for each type of stay in the hospital and each clinical department requesting a radiologic examination for such patients has been counted.
- For the same two types of diseases the number of patients with repeated examinations using the same or different modalities was counted.
- For two clinical departments requesting radiologic examinations the time between the request and the availability of the radiologic report has been evaluated.

#### Reports for Hospital B

Each of the following reports was requested for each site of the hospital as well as for regular radiology and neuroradiology:

- The number of patients, appointments, and examinations for each modality was counted. In separate reports, these numbers were further split by each shift and hour during the day or the body regions listed for the next report.
- For repeated examinations using the same or different modalities for the same body region, the number of patients was counted. The body regions of interest for this hospital were the abdomen, the cervical spine, the thoracic spine, the lumbar spine, the ankle joint, the knee joint, the hip joint, the shoulder joint, and the liver. In another report, the total time for these sequences of modalities was evaluated.
- For each modality, the time between the request for an examination and the actual start of it, as well as the availability of the radiologic report, was evaluated. In a similar way, the duration for just the examination itself was evaluated.
- The use of each radiologic device was evaluated by the duration for just the examination itself as well as the duration from the start of an examination until the start of the next examination using the same device.
- For another report, the number of patients at the radiology department for the first time or using each modality for the first time during a year was counted.
- The number of examinations was also counted for patients not older than 18 years and for the following special treatments: osteodensitometry, teleradiology, mammography, and nuclear medicine.
- Specifically for radiologic examinations of the spine, the difference between the actual time a patient stayed in the hospital in comparison to the time recommended by a clinical guideline was evaluated.

#### Reports for Hospital C

- The number of appointments and examinations for each modality was counted for each site of the hospital and each year. These numbers were calculated separately for each hospital department requesting a radiologic examination, for each shift during the day, for each type of stay in the hospital, and for each type of insurance a patient has.
- Only the number of MRI examinations was calculated for each hospital site and year separated by the following body regions: spine, abdomen, upper abdomen, pelvis, small intestine, joints, soft tissues of the neck, hand, foot, chest, skull, shoulder, and heart.

#### Reports for Hospital D

- For each site of the hospital, each year, type of stay in the hospital, and shift, the number of appointments and examinations was counted for each modality.
- Because for this hospital, the names of external private medical practices requesting radiologic examinations were also provided, the number of appointments and examinations requested by each of them was also counted for each hospital site, year, and modality.

With these reports, all the requests of hospitals A and B and some of the requests of hospitals C and D could be fulfilled. As mentioned before, hospitals C and D were not able to provide patient identifiers along with the rest of their exported data, and therefore, no number of patients and no sequences of multiple examinations (as they usually do not occur during the same appointment) could be calculated. The data provided by hospitals C and D also contained no time stamps except the time an examination started, and therefore, no time differences between, for example, the request for an examination and the actual appointment or the availability of the radiologic report, could be evaluated.

However, as long as the hospitals were able to provide the necessary data, all their requests could be fulfilled, and therefore, all of them were satisfied with the developed system.

## Discussion

### Principal Findings: Used Data

As shown by the numbers in the previous section, the imported data was diverse, with different numbers of years and attributes. The number of generated values was different as well (depending on the requested reports). However, some hospitals were not able to provide all the data for their requested reports, such as hospitals titled C and D, which could not (or only with a lot of effort) provide a patient identifier, which resulted in the inability to create any reports with counts of patients. Nevertheless, due to its configurable and modular approach, the developed system can be used for these RIS exports as well, only requiring the creation of a new configuration file as well as some implementations of the interfaces for additional calculations. For hospital B, by far, the maximum number of reports was created, which resulted in the number of generated values exceeding the number of imported ones. One of these calculations, for example, was to count how many patients encountered multiple examinations for the same medical question with the same or different modalities. This directly pointed out multiple cases in which, for example, X-ray examinations had been conducted, followed by a CT or MRI examination, where only a CT or MRI examination would have been necessary, resulting in unnecessary radiation exposure for the patients as well as unnecessary radiology appointments.

### Process of Importing the Radiology Data Into the Data Warehouse

When comparing the different numbers related to the import process, we found a correlation between the number of generated and calculated values and the time the developed tool needed to process them. However, even the RIS export with the maximum imported and calculated values (of hospital B) needed only about 1 hour 10 minutes for the whole process, making it fast to use even when installed in a new environment. For most reports, this time is only needed once, and multiple different reports can be created with the system afterward. If adaptions are needed (like for additional calculations during the import process), the mechanism for incremental updates can be used so that the time until the adaptions can be used for reports is even shorter. The storage required for the database and the Solr index together (31 GB for the largest data set of hospital B) can be easily found on many existing systems, and therefore, in most cases, no additional drives need be bought when using this tool. As shown in Table 2, the sizes of the database and Solr index were nearly identical for 3 of the hospitals. The difference between these sizes for hospital C was the reason that the data provided by it as well as the calculated values were mostly Boolean values. Although they are saved in similar form as other types of values in the database, the Solr index does not need to save and process any concrete textual or numeric value for them, resulting in the Solr index being a lot smaller than the database.

### Creating Reports on the Radiology Data With the Data Warehouse

For many kinds of reports, the developed system can calculate and export them in a few seconds, as shown in Table 3. This allows a user to quickly iterate and try multiple configurations of a query. By using the preview option of PaDaWaN’s interface, intermediate results do not always have to be exported to Excel or CSV. Even the third report in Table 3 could be created in less than 1.5 minutes for all 4 hospitals, although many single Solr queries were necessary for them. In all these reports, the queries were similar, resulting in the average time for each query becoming shorter with the total number of queries. Another observation from the created reports is that with a larger Solr index (hospital B has the largest one), the average time for each Solr query more than doubles compared to the reports created for the other hospitals but still goes down to 25 ms during the creation of the third (and largest) report.

### Comparison With the Creation of Reports Directly in Excel

When comparing the developed tool with report creation directly in Excel, except for the combination of data from multiple Excel files into 1 (which is not necessary for the developed tool, because the RIS data are combined into 1 database and Solr index), no import of data are required, making this step faster and easier in Excel. However, for the calculation of additional values depending on the type of calculation, the required Excel formulas can get quite complex and are therefore more difficult to develop and maintain compared to a calculation written using the Kotlin method. To circumvent this disadvantage, Excel’s ability to add scripts [28] can be used. During the creation of reports, the main disadvantage of using only Excel is the requirement for complex formulas, making the whole report more difficult to create and maintain compared to configuring a query in PaDaWaN’s web interface. Especially the addition/deletion of an attribute to/from any row, column, or filter is easy in PaDaWaN’s GUI, while this requires a user to adapt the Excel formulas in every cell. Therefore, PaDaWaN allows for easy ad hoc adaptation of reports even while discussing them with clinicians. Such ad hoc adaptation also benefits from the fast execution times of most PaDaWaN queries, as explained before. The drawbacks resulting from the use of Excel formulas for creating reports can be partially overcome by using Excel’s feature to create pivot tables. This allows a user, in a similar way to PaDaWaN, to configure rows, columns, and filters of the requested table as well as the kind of numbers (eg, counts of patients or examinations) that should be returned. The disadvantages of this feature are that no logical combinations of attributes for a single column, row, or filter can be specified and would require additional precalculated columns. It also lacks PaDaWaN’s ability for advanced searches on textual data directly as part of the query [18]. Altogether this evaluation showed that although most kinds of reports can be somehow created with Excel, especially more complex queries are difficult to configure and maintain there, while this can be done easily in PaDaWaN’s GUI. A limitation of the developed tool is the requirement for an initial setup and for additional training of the clinicians on how to use it, while Excel is a tool that is already installed in many hospitals and many clinicians are already familiar with its usage.

### Comparison With Alternative Solutions

In addition to the creation of reports directly in Excel, other solutions already exist that provide a user with key metrics on medical data. A comparison of the solutions introduced in the State of the Art section is presented in Table 4.

**Table 4:** Comparison of different existing solutions to calculate key metrics of radiology data (or of medical data in general in the last column).

Solution	Studies					
	[6-9]	[10]	[11]	[12]	[13,14]	[15,16]
Graphical results	Yes	No	Yes	Yes	No	Yes
Graphical query definition	No	Limited	Limited	Limited	No	Yes
User-defined queries	No	Limited	Limited	Limited	Yes	Yes
Additional calculations during import	N/A^a^	No	No	No	No	No
Import independent of a specific RIS^b^	No	No	Yes	No	No	Yes

^a^N/A: not applicable. Because these solutions operate directly on a radiology information system (RIS), no intermediate storage is used and therefore no additional attributes can be saved to it.

^b^RIS: radiology information system.

The first 4 solutions provide dashboards for the following uses cases: general imaging use [6], ordered and performed imaging studies for the emergency department [7], scheduled and in-progress examinations in pediatric radiology [8], and various metrics on orders, acquisition, interpretation, and reporting of radiologic images [9]. Although all of them could present their results as graphics, no additional queries (in addition to the ones predefined for the graphics) could be performed. Furthermore, the solutions only work with 1 or multiple specific RISs.

The next 3 solutions work in a different way, as their primary purpose is to display information about individual patients who are currently treated (or about to be treated) in the radiology department [10-12]. The values shown by them can be filtered, for example, by a specific type of examination, but no real queries on these data can be defined by the user. The graphics provided by Henkel et al [11] are limited to single patients and show, for example, the history of 1 of their laboratory values. Munbodh et al [12] also provide predefined graphics for the total number of examinations of different kinds during the past month. Although they all use intermediate storage for all the patients’ data from the hospitals and RISs, no additional calculations on these data can be performed during the import, and of the 3, only the solution provided by Henkel et al [11] is not tied to a specific hospital or RIS.

The next 2 solutions use a data warehouse as a business intelligence tool for the radiology department [13] and combine radiology with pathology data [14]. Although both solutions allow for user-defined queries to be executed, these queries must be specified using Excel or SQL and not via a GUI. They also cannot create graphics from the query results and are tied to a specific RIS. Additionally, no precalculations on the RIS data can be performed and saved in the data warehouse.

The last 2 solutions are importers for i2b2 [15] and for i2b2 as well as PaDaWaN [16] and are not dependent on a specific RIS. They can use all the capabilities of these data warehouse solutions, including the ability for user-defined queries via a GUI and to show some predefined graphics based on these queries. However, because no additional calculations can be performed and saved to the data warehouse during the import, some kinds of reports are difficult or even impossible to create (eg, the evaluation of repeated examinations of the same body region). The developed system has the capability of additional calculations and therefore supports the most diverse kinds of reports.

### Verification of the Calculated Metrics

As mentioned in the Introduction section, there are 2 main purposes of calculating all these metrics: From a patient’s perspective, the waiting time for an appointment in the radiology department should be as short as possible and the exposure to radiation should be as low as possible. The hospital, however, wants to maximize its profits. By reducing the time until a diagnosis has been made, the patients can stay in the hospital for fewer days, which therefore allows the hospital to treat more patients. In addition, by eliminating or at least reducing unnecessary repeated examinations of the same region of the body, the radiology department also has the capacity to treat more patients. Treating more patients results in more profit for the hospital and shorter waiting times for patients. The reduction in unnecessary examinations also lowers the patients’ exposure to radiation. Thus, both main purposes of the developed system can be achieved if metrics concerning the waiting times for appointments and diagnosis as well as repeated examinations can be calculated. The way this can be done has already been described before. For a hospital to be able to improve these metrics, it is also important that potential reasons for longer waiting times and repeated examinations be evaluated. Just from the RIS data themselves, this is, for example, possible by comparing the use of different radiologic devices to check whether the purchase of additional devices is necessary. Another possibility is the comparison of different hospital departments that request radiologic examinations. If a metric is significantly worse for one department compared to others, maybe the communication between this department and radiology needs to be improved. However, the developed system is not limited to RIS data alone. By combining these data with other data from the hospital information system in the data warehouse, additional possibilities for improvements can be found. For example, by comparing the diagnosis made by the radiology department with the final clinical diagnosis, the quality of the radiologic diagnosis can be evaluated. In addition, because the data warehouse can store data from multiple years, along with their time stamps, the developed system also supports the verification if any measure taken results in the desired improvement of specific metrics.

### Limitations of and Bias in the Calculated Metrics

As all the metrics calculated by the developed system are based on data from the RIS and the hospital information system, their quality directly depends on the quality of the hospital’s documentation. As this is not verifiable by the developed tool, it could only be assumed that this is done correctly. As mentioned in the previous paragraph, it is also important for the developed system that potential reasons for, say, longer waiting times for an appointment be evaluated. If, for example, a difference between the radiologic diagnosis and the final clinical diagnosis is not documented, it cannot be checked as a potential reason for longer waiting times or unnecessary examinations. In general, inferring conclusions from the calculated metrics can be difficult if potential causes are not documented. For example, if for a medical question, the ideal radiologic examination would be MRI, it may not be conducted, because it is too expensive or enough devices are not available. This could, for example, be further validated by comparing the performed examination with the one recommended by clinical guidelines (like the ones provided by the German Radiation Protection Commission [29]). Another potential bias in the calculated metrics may result from radiologic examinations that are performed externally (eg, at a private medical practice). If not properly documented, this is a missed indicator for the need for additional devices or employees. By combining RIS data with additional data from the hospital in the data warehouse, the text search capabilities of the data warehouse can potentially be used to find information about such external examinations in the patient’s discharge letter. Additionally, the interpretation of the metrics could be different among different hospitals, resulting in limitations of the comparability of the metrics between them. Although, for example, a waiting time of a few days for an appointment in the radiology department could be acceptable for one hospital, it could be inacceptable for another one.

### Conclusion

To summarize, the developed system has its main advantages in being generic concerning the export of different RISs as well as concerning the configuration of queries for various reports. To use it, the only requirement is the ability of an RIS to create an Excel, CSV, or JSON export. This can then be imported by creating a simple configuration file in Excel or as a CSV file as well. During the import process, additional values can be calculated by implementing several provided interfaces. If further values should be added later, this is easily possible with the ability to use incremental updates. Various reports with any combination of and restriction on the imported attributes can then be graphically configured using PaDaWaN’s web interface. Finally, the results of these reports can be exported into the standard formats Excel and CSV so that they can be easily processed with many different tools.

The whole tool as a Docker [30] image, a sample RIS export, and a configuration file are publicly available on PaDaWaN’s website [31].

The developed tool can in the future be further enhanced by, for example, adding the ability to calculate other numbers than the count of patients, appointments, or examinations, such as the average of numeric values found by a query. To improve the presentation of the results, the current ability to create graphics for some predefined reports can also be extended to be configurable by a user and therefore allow for the creation of many kinds of graphical reports.

## Abbreviations

CT: computed tomography
GUI: graphical user interface
JSON: JavaScript Object Notation
MRI: magnetic resonance imaging
MXQL: Medical XML Query Language
PaDaWaN: Patient Data Warehouse Navigator
RIS: radiology information system

## References

[1] Georgiana V, Kartawiguna D (2016). Evaluation of radiology data warehouse implementation on education, research, and quality assurance.

[2] Karami M, Safdari R (2017). From information management to information visualization: development of radiology dashboards. Appl Clin Inform.

[3] Karami M (2014). A design protocol to develop radiology dashboards. Acta Inform Med.

[4] Karami M, Safdari R, Rahimi A (2013). Effective radiology dashboards: key research findings. Radiol Manag.

[5] Mansoori B, Novak RD, Sivit CJ, Ros PR (2013). Utilization of dashboard technology in academic radiology departments: results of a national survey. J Am Coll Radiol.

[6] Halpern DJ, Clark-Randall A, Woodall J, Anderson J, Shah K (2021). Reducing imaging utilization in primary care through implementation of a peer comparison dashboard. J Gen Intern Med.

[7] Scheinfeld MH, Feltus W, DiMarco P, Rooney K, Goldman IA (2020). The emergency radiology dashboard: facilitating workflow with realtime data. Curr Probl Diagn Radiol.

[8] Shailam R, Botwin A, Stout M, Gee MS (2018). Real-time electronic dashboard technology and its use to improve pediatric radiology workflow. Curr Probl Diagn Radiol.

[9] Nagy PG, Warnock MJ, Daly M, Toland C, Meenan CD, Mezrich RS (2009). Informatics in radiology: automated web-based graphical dashboard for radiology operational business intelligence. Radiographics.

[10] Burns JL, Hasting D, Gichoya JW, McKibben B, Shea L, Frank M (2020). Just in time radiology decision support using real-time data feeds. J Digit Imaging.

[11] Henkel M, Horn T, Leboutte F, Trotsenko P, Dugas SG, Sutter SU, Ficht G, Engesser C, Matthias M, Stalder A, Ebbing J, Cornford P, Seifert H, Stieltjes B, Wetterauer C (2022). Initial experience with AI Pathway Companion: evaluation of dashboard-enhanced clinical decision making in prostate cancer screening. PLoS One.

[12] Munbodh R, Roth TM, Leonard KL, Court RC, Shukla U, Andrea S, Gray M, Leichtman G, Klein EE (2022). Real-time analysis and display of quantitative measures to track and improve clinical workflow. J Appl Clin Med Phys.

[13] Prevedello LM, Andriole KP, Hanson R, Kelly P, Khorasani R (2010). Business intelligence tools for radiology: creating a prototype model using open-source tools. J Digit Imaging.

[14] Rubin DL, Desser TS (2008). A data warehouse for integrating radiologic and pathologic data. J Am Coll Radiol.

[15] Bauer C, Ganslandt T, Baum B, Christoph J, Engel I, Löbe M, Mate S, Prokosch H, Sax U, Stäubert S, Winter A (2015). The Integrated Data Repository Toolkit (IDRT): accelerating translational research infrastructures. J Clin Bioinformatics.

[16] Fette G, Kaspar M, Dietrich G, Ertl M, Krebs J, Stoerk S, Puppe F (2017). A customizable importer for the clinical data warehouses PaDaWaN and I2B2. Stud Health Technol Inform.

[17] Liman L, Fette G, Krebs J (2021). Calculating key figures for radiology departments using a clinical data warehouse ? A technical case report. Stud Health Technol Inform.

[18] Dietrich G, Krebs J, Fette G, Ertl M, Kaspar M, Störk S, Puppe F (2018). Ad hoc information extraction for clinical data warehouses. Methods Inf Med.

[19] Introducing SQL Server 2022. Microsoft.

[20] MySQL HeatWave - one MySQL database service for OLTP, OLAP, and ML. MySQL.

[21] Dinu V, Nadkarni P (2007). Guidelines for the effective use of entity-attribute-value modeling for biomedical databases. Int J Med Inform.

[22] International Classification of Diseases, Tenth Revision, Clinical Modification (ICD-10-CM). Centers for Disease Control and Prevention.

[23] Learn more about Solr. Apache Software Foundation.

[24] Abfragesprache – Lehrstuhl für Künstliche Intelligenz und Wissenssysteme. Institut für Informatik - Universität Würzburg.

[25] Kotlin v1.8.21. Kotlin Foundation.

[26] Apache Solr Reference Guide: updating parts of documents. Apache Software Foundation.

[27] Apache Tomcat. Apache Software Foundation.

[28] Differences between Office Scripts and VBA macros. Microsoft.

[29] (2020). Recommendations for medical imaging procedures. Strahlenschutzkommission.

[30] Develop faster. Run anywhere. Docker Inc.

[31] Download – Chair of Computer Science VI – artificial intelligence and applied computer science. Institut für Informatik - Universität Würzburg.

